# The chaperonin CCT inhibits assembly of α-synuclein amyloid fibrils by a specific, conformation-dependent interaction

**DOI:** 10.1038/srep40859

**Published:** 2017-01-19

**Authors:** Begoña Sot, Alejandra Rubio-Muñoz, Ahudrey Leal-Quintero, Javier Martínez-Sabando, Miguel Marcilla, Cintia Roodveldt, José M. Valpuesta

**Affiliations:** 1 Departamento de Estructura de Macromoléculas, Centro Nacional de Biotecnología (CNB-CSIC), Campus de la Universidad Autónoma de Madrid, 28049 Madrid, Spain; 2 Fundación IMDEA-Nanociencia, Campus de la Universidad Autónoma de Madrid, 28049 Madrid, Spain; 3 Unidad de Proteómica, Centro Nacional de Biotecnología (CNB-CSIC), Campus de la Universidad Autónoma de Madrid, 28049 Madrid, Spain; 4 Andalusian Center for Molecular Biology and Regenerative Medicine (CABIMER)-Spanish National Research Council (CSIC), Universidad Pablo de Olavide, University of Seville, Seville, Spain

## Abstract

The eukaryotic chaperonin CCT (chaperonin containing TCP-1) uses cavities built into its double-ring structure to encapsulate and to assist folding of a large subset of proteins. CCT can inhibit amyloid fibre assembly and toxicity of the polyQ extended mutant of huntingtin, the protein responsible for Huntington’s disease. This raises the possibility that CCT modulates other amyloidopathies, a still-unaddressed question. We show here that CCT inhibits amyloid fibre assembly of α-synuclein A53T, one of the mutants responsible for Parkinson’s disease. We evaluated fibrillation blockade in α-synuclein A53T deletion mutants and CCT interactions of full-length A53T in distinct oligomeric states to define an inhibition mechanism specific for α-synuclein. CCT interferes with fibre assembly by interaction of its CCTζ and CCTγ subunits with the A53T central hydrophobic region (NAC). This interaction is specific to NAC conformation, as it is produced once soluble α-synuclein A53T oligomers form and blocks the reaction before fibres begin to grow. Finally, we show that this association inhibits α-synuclein A53T oligomer toxicity in neuroblastoma cells. In summary, our results and those for huntingtin suggest that CCT is a general modulator of amyloidogenesis via a specific mechanism.

α-synuclein (α-syn) is an intrinsically disordered, 140-residue protein that is expressed extensively in neurons (1% of total cytosolic proteins)^1^. Although its exact function remains to be defined, it is proposed to participate in synaptic vesicle release and trafficking, fatty acid binding, and regulation of enzymes, neurotransmitters and vesicles^2^. α-syn is the principal component of the Lewy bodies found in some neurological diseases termed synucleinopathies, such as Parkinson’s disease (PD), multiple system atrophy (MSA), dementia with Lewy bodies (DLB), neurodegeneration with brain iron accumulation (NBIA) and pure autonomic failure (PAF)^3^. Three point mutations (A30P, E46K or A53T) or the overexpression of wild-type (wt) α-synuclein, which are known to promote oligomerization and amyloid fibre formation, are associated to early onset Parkinson’s disease. This aggregation induces neuron death, although there is some debate as to whether the fibres or the soluble oligomers formed during the fibrillation reaction are the main cytotoxic species. There is nonetheless a consensus that the study of proteins or molecules that modulate wt or mutated α-syn aggregation is essential for future therapies.

Molecular chaperones are central to protein homeostasis, as they are involved not only in folding of proteins, but also in their degradation and in preventing their aggregation^4^, as well as in the inhibition of α-syn aggregation and toxicity^5^. The effect of the eukaryotic chaperonin CCT on α-syn aggregation nonetheless remains unaddressed, despite the fact that quantitative proteomics has identified CCT as a α-syn-associated protein^6^,^7^. Like most chaperonins, CCT is built of two back-to-back rings, each formed by eight different, homologous subunits^8^. Each subunit is constituted of three domains; the equatorial domain hosts the ATP-binding site and most intra- and inter-ring contacts, the apical domain hosts the substrate binding sites, and the intermediate domain acts as a link between these two domains and transmits signals generated in the equatorial domain to the apical domain after ATP binding and hydrolysis^9^.

Although originally described as a specific folder of the cytoskeletal proteins actin and tubulin, it has become clear that CCT is involved in assisting the folding of a larger subset of proteins, some with key roles in essential processes such as cell signalling and cell cycle control^10^. Furthermore, CCT inhibits amyloid fibre assembly and toxicity of the poly-Q extended mutant of huntingtin, the protein responsible for Huntington’s disease^11^,^12^,^13^. This raises the possibility that CCT can modulate other amyloidopathies. Quantitative proteomics identified CCT as an α-syn-associated protein^6^,^7^ when amyloid protein aggregation was induced by rotenone in dopaminergic neuronal MES cells. In addition, the neuroprotective functions of Sir2, a CCT-activating protein with roles in aggresome segregation in an α-syn-based yeast model of PD, are suggested to be CCT-mediated^14^. The direct effect of CCT on α-syn fibrillation has nonetheless remained unaddressed. Here we show that CCT blocks the *in vitro* fibrillation and toxicity of α-syn A53T, a mutant responsible for Parkinson’s disease, and provides biochemical and structural insight into a specific mechanism. These results suggest that CCT is a general modulator of amyloidogenesis via an amyloid protein-specific mechanism.

## Results

### CCT inhibits α-syn A53T fibrillation

**Figure 1:**
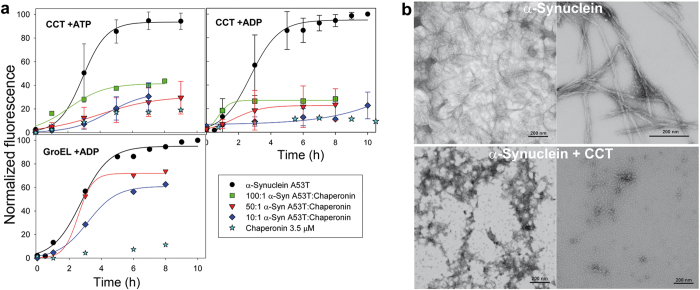
CCT inhibits α-syn A53T fibrillation. (**a**) Fibrillation kinetics of α-syn A53T in the presence of ATP or ADP and increasing molar ratios of CCT or GroEL, measured by ThT fluorescence. Chaperonins without α-syn A53T were used as controls. Fluorescence data is normalized as a percentage of maximum fluorescence. Bars show mean ± SD, *n* = 3–8. For GroEL, one representative experiment is shown. (**b**) TEM images of the final point of fibrillation kinetics for α-syn A53T, alone or with CCT and ADP.

To study α-syn A53T fibrillation, we used a thioflavin T (ThT) fluorescence assay. ThT binds specifically to amyloid fibres, after which its fluorescence increases. In our experimental conditions, α-syn A53T formed fibres over a 10 h timescale, following typical nucleation-dependent kinetics^15^ (Fig. 1a). A lag phase corresponding to the nucleation step was followed by a fibre elongation step, until the reaction reached a plateau; fibres were then visualized by negative staining transmission electron microscopy (TEM) (Fig. 1b). To test whether the chaperonin CCT inhibits α-syn A53T amyloid fibre assembly, we evaluated the CCT effect on α-syn A53T fibrillation in the presence of ATP or ADP (Fig. 1a). An ATP regenerative system could not be used due to pyruvate kinase instability in shaking conditions. Nevertheless, as CCT ATPase activity at 37 °C is 6.8 μM ATP/min^−1^μM^−1^ CCT, the 10 mM ATP added is sufficient to support CCT ATPase activity throughout the experiment. Nucleotide-free chaperonin was not tested, as this situation is not found in a physiological context. The ability of CCT to refold protein substrates relies on ATP binding and subsequent hydrolysis^10^, whereas in the presence of ADP, the chaperonin can interact with substrates but not refold them. CCT prevented α-syn A53T fibre assembly in the presence of both nucleotides, even at substoichiometric concentrations (1:100 CCT:α-syn A53T monomer) (Fig. 1a). These data suggest an inhibitory effect of CCT due to its α-syn A53T binding rather than to active folding. TEM images supported the fluorescence experiments, and showed that mostly amorphous aggregates formed in the presence of CCT (Fig. 1b), although short fibres and round aggregates were also found. GroEL, the prokaryotic CCT counterpart, had a much weaker effect on α-syn A53T fibre formation than the eukaryotic chaperonin, even at a molar ratio of 1:10 GroEL:α-syn A53T (Fig. 1a), which indicates a more specific CCT effect in blocking α-syn A53T aggregation. CCT also prevented fibrillation of wt α-syn, which showed that the CCT effect is not specific to the A53T mutation (Supplementary Fig. S1).

### CCT inhibition of α-syn fibre formation is induced by interaction with its NAC region

**Figure 2:**
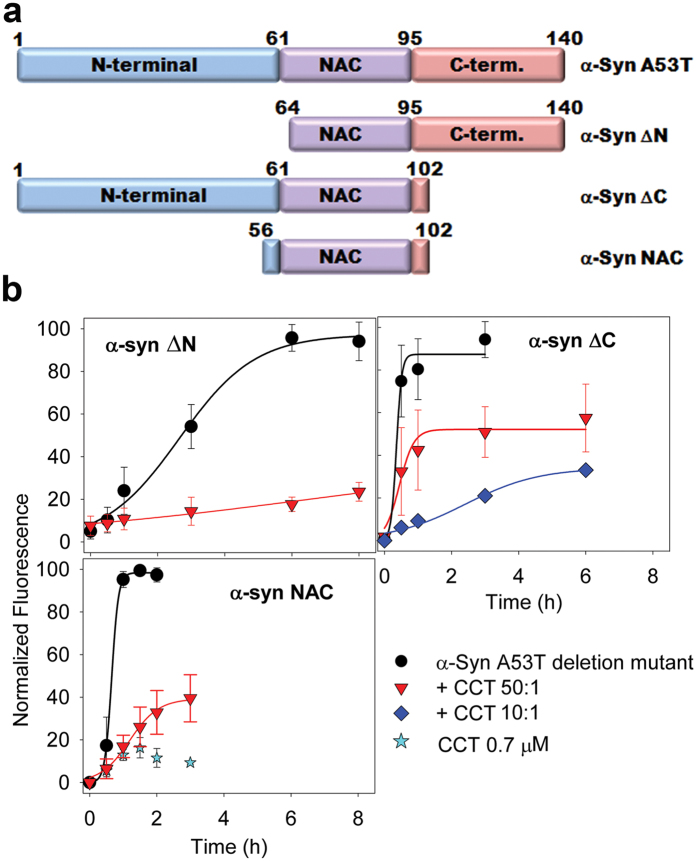
Effect of CCT on fibrillation of α-synuclein A53T deletion mutants. (**a**) Scheme of full-length α-syn A53T and the deletion mutants used for fibrillation experiments. (**b**) Fibrillation kinetics of the α-syn A53T deletion mutants in the presence of ADP and different molar ratios of CCT, measured by ThT fluorescence. As a control, the fluorescence of CCT in the absence of α-syn A53T was also tested.

Once we had established that specific CCT interaction with α-syn A53T inhibits fibre formation, we tested which α-syn regions were involved in CCT interaction. α-syn consists of (1) an amphiphilic, basic N-terminal region, with helical propensity^16^,^17^ (Fig. 2a), (2) a central hydrophobic region (non-Aβ component; NAC) responsible mainly for β-sheet propensity and fibrillation, and (3) a very negatively charged, unstructured C-terminal region. Tam *et al*. showed that CCT inhibits huntingtin fibrillation by interacting with the N-terminal amphipathic α-helix of the amyloid protein^18^. The α-syn N-terminal and central parts (residues 3 to 95) form two amphipathic α-helices after interaction with lipids^19^, and although α-syn is mostly unfolded in solution, the 15–31 and 42–64 segments in the N-terminal region (Fig. 2a) are prone to helix formation^16^,^17^.

Based on this information, we constructed three α-syn A53T deletion mutants (Fig. 2a), α-syn ΔN (lacking the N-terminal region), α-syn ΔC (lacking most of the C-terminal part) and α-syn NAC (lacking most of the N- and C-terminal parts). The mutants were overexpressed, purified, and tested for fibre formation and the CCT effect on their oligomerization. The α-syn ΔN deletion mutant induced fibre formation (Fig. 2b), and removal of the N-terminal part increased the CCT inhibitory effect on fibre assembly (Fig. 2b), which suggests that this region is not the main CCT binder. Although CCT interacts with the N-terminal amphipathic helix of huntingtin^18^, the chaperonin does not bind α-syn regions with propensity to form amphipathic helices, which indicates that the CCT interaction regions in these proteins differ structurally. Deletion of the acidic C-terminal region (α-syn ΔC, Fig. 2a) accelerated fibre assembly (Fig. 2b)^20^,^21^. In α-syn monomers, the C terminus interacts with the N-terminal and the NAC regions, burying the aggregation-prone area and inhibiting fibre formation. Deletion of the C-terminal region thus appears to expose the NAC domain, which accelerates fibrillation^22^,^23^. Although CCT interfered with α-syn ΔC fibre assembly, the degree of inhibition was less than that of full-length α-syn A53T. In the presence of ADP, CCT decreased the percentage of α-syn A53T fibres up to 11 and 23% at molar ratios of 1:10 and 1:50, respectively. In the case of α-syn ΔC, these values were 33 and 58% (compare Figs 1a and 2b). The isolated NAC domain (α-syn NAC) formed fibres more rapidly (Fig. 2b), which clearly indicated that it is the most important region in this process. CCT inhibited the α-syn NAC fibrillation more than that of the α-syn ΔC (38% vs. 58% at a molar ratio of 1:50 CCT:deletion mutant), but inhibition was lower than of full-length and the α-syn ΔN. These results indicate that the α-syn NAC region is mainly responsible for interaction with CCT, although the C-terminal part also appears to be involved.

### The CCT cavity is involved in the interaction with α-syn A53T pre-fibrillar oligomers

Our data suggested that CCT inhibits α-syn A53T fibril assembly by interacting mainly with the NAC region. To elucidate the mechanism that underlies this inhibition, we determined which α-syn A53T species produced during fibrillation (monomers, pre-fibrillar oligomers or fibres) interact with CCT. Most previously studied chaperones interact with more than one α-syn species; for example, Hsp70 interacts with monomers, pre-fibrillar oligomers and small fibres^24^, whereas Hsp90 interacts with monomers^25^ and pre-fibrillar oligomers^25^,^26^. CCT impairs huntingtin fibrillation by binding to the aggregation-prone N-terminal of monomers, but also by capping the tips of small fibres with its apical domain, thus inhibiting their elongation^11^,^27^.

**Figure 3:**
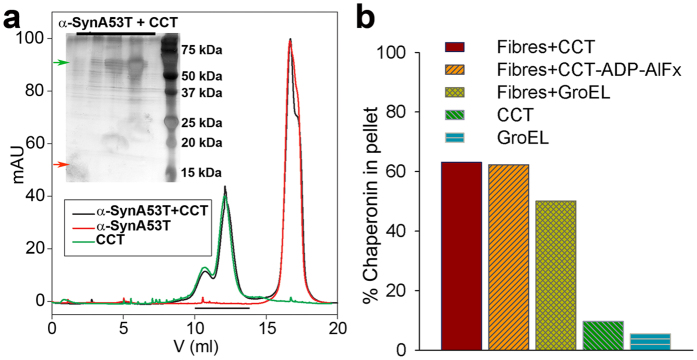
CCT binding to α-syn A53T monomers and fibres. (**a**) CCT binding to α-syn A53T monomers. SEC profile of CCT, α-syn A53T and the mixture of both. Inset: SDS-PAGE of the fractions corresponding to the CCT peak (black line in SEC profile) of the α-syn A53T + CCT sample. CCT subunit band, green arrow. No α-syn A53T band is visible in the gel, which should appear near the 15 kDa position (red arrow) (see Fig. 4a). (**b**) CCT binding to fibres assayed by ultracentrifugation. Percentage of CCT, CCT with ADP-AlF_3_ or GroEL that coprecipitate with fibres. The percentage of CCT or GroEL precipitated in the absence of fibres is shown as a control.

To test its binding to α-syn A53T monomers, we incubated CCT with a monomer excess to promote interaction. Complex formation was tested by size exclusion chromatography (SEC) on Superose 6 resin (Fig. 3a). As a control, CCT and α-syn A53T were loaded individually; they showed completely different elution profiles, as predicted by their very different molecular mass (~1 MDa for CCT, 15 kDa for α-syn A53T). When CCT and α-syn A53T were pre-incubated and loaded onto the SEC column, the elution profile was that of a mixture of these two molecules. SDS-PAGE (sodium dodecyl sulphate-polyacrylamide electrophoresis) analysis of the CCT peaks did not show α-syn A53T (a ~15 kDa band, see Fig. 3a), which indicates that the chaperonin does not interact with the α-syn A53T monomers. This observation is supported by CCT inhibition of α-syn A53T fibrillation at 1:100 (substoichiometric concentration; Fig. 1a), and implies chaperonin binding to α-syn A53T high-order multimers.

CCT interaction with fibres purified from the elongation reaction (see Methods) was analysed by ultracentrifugation (Fig. 3b). The fibres, but not CCT or GroEL alone, sedimented in the centrifugation conditions used for fibre isolation (141,000 xg, 1 h) (see Methods). When incubated with fibres, a large percentage of the CCT (up to 60%) cosedimented with them. To confirm the specificity of this interaction, we used two controls, GroEL, and a closed-lid version of CCT generated by incubation of the chaperonin with the non-hydrolysable ATP analogue ADP-AlF3^28^. The prokaryotic chaperonin GroEL interacted with the α-syn A53T fibres to a similar extent as CCT (Fig. 3b). As GroEL blocked α-syn A53T fibre formation to a lesser degree than CCT, this result calls into question the specificity of the interaction between CCT and α-syn A53T fibres. Elongation of the huntingtin fibre is inhibited by its interaction with the region of the CCT apical domain located inside the cavity, the substrate-binding region^27^. As a second control, we tested whether this is also the case for CCT and α-syn A53T by incubating CCT with ADP-AlF3, which mimics the ATP hydrolysis intermediate and induces closure of the chaperonin lid, thus blocking the CCT cavity and the substrate-binding sites from the exterior^28^ (Supplementary Fig. S2). The closed CCT also interacted with fibres (Fig. 3b), which strengthens the idea that the interaction between CCT and the α-syn A53T fibres is not functional. It also allows speculation that α-syn fibre interaction with CCT induces chaperonin depletion, to generate a negative effect in protein homeostasis as suggested for Hsp70^29^.

**Figure 4:**
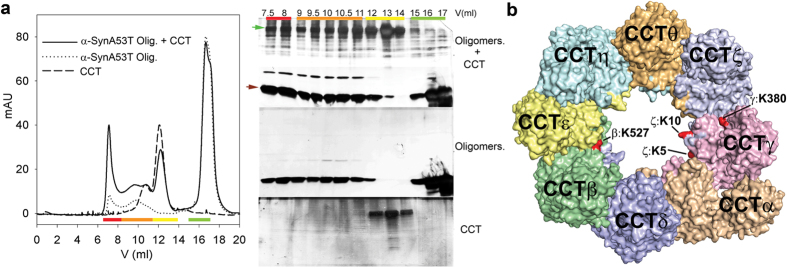
CCT binding to soluble α-synuclein A53T oligomers. (**a**) Left, SEC analyses of CCT, α-syn A53T oligomers and the mixture of both. Coloured lines at bottom mark the different peaks. Right, SDS-PAGE of the samples eluted in the left panel, after silver staining, colour-coded as in the elution profile. Red and green arrows indicate bands corresponding to CCT subunits and to α-syn A53T monomers, respectively. (**b**) XL-MS of the crosslinked CCT-α-syn A53T soluble oligomer complex. Top view of CCT atomic structure (pdb ID code 4B2T) showing residues identified as crosslinked to α-syn A53T (red).

To test the CCT interaction with soluble oligomers of α-syn A53T, we prepared pre-formed oligomers^30^ and analysed them by TEM (Supplementary Fig. S3). We observed heterogeneous structures, which were round, ring-shaped or elongated, with a maximum width of ~10 nm. The absence of monomers was confirmed by dynamic light scattering (DLS; Suplementary Fig. S4). The oligomers were loaded onto a Superose 6 10/30 column, alone or with CCT (Fig. 4a). In the former case, the soluble oligomers eluted in two main peaks (Fig. 4a, red and orange bars), which correlates with two populations, large and small, of soluble α-syn A53T oligomers, similar to the results for wt α-syn of Lorenzen *et al*.^31^; a third peak (Fig. 4a, green bar) that corresponded to monomeric α-syn was probably due to partial oligomer solubilisation by dilution in the column. SDS-PAGE analysis of the three peaks showed a band that corresponded to α-syn A53T. The mixture of α-syn A53T oligomers with CCT yielded several peaks, one for CCT (Fig. 4a, yellow bar; similar to that of control CCT) and the two peaks for α-syn A53T oligomers (Fig. 4a, red and orange bands). SDS-PAGE analysis of these peaks showed that some CCT co-eluted with the large and small oligomers, which indicated stable CCT interaction with soluble α-syn A53T oligomers.

We also analysed the CCT-α-syn A53T oligomer complex in the presence of ATP (Supplementary Fig. S5). Although we showed that ATP is not necessary for α-syn A53T fibrillation inhibition by CCT, it is possible that the CCT folding cycle driven by its ATPase activity modifies oligomer size. We tested oligomer size using SEC (Supplementary Fig. S5a) and acrylamide native gels (Supplementary Fig. S5b), and showed that CCT in the presence of ATP did not modify oligomer size or increase monomer amounts in solution.

To test whether these soluble oligomers interact with CCT as substrates, that is, through regions within the CCT cavity, we treated a mixture of CCT and α-syn A53T soluble oligomers with the two-armed lysine/serine crosslinker DTSSP (3,3′-dithiobis (sulphosuccinimidylpropionate)); the complex was purified by SEC and studied by crosslinking mass spectrometry (XL-MS). We showed that the NAC region is the CCT interaction site; this region hosts only a single lysine residue, K80, and we therefore did not anticipate obtaining specific interacting residues. Since DTSSP is 12 Å long, we consider that the lysines and serines must be at approximately this distance to be crosslinked. We were thus able to obtain information about neighbouring regions surrounding the contact site.

**Figure 5:**
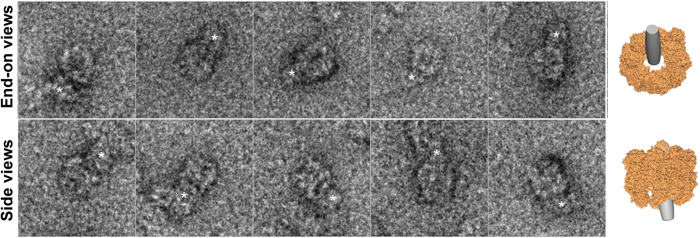
TEM views of CCT-α-syn A53T oligomers. Gallery of representative end-on (top) and side (bottom) views of the complexes eluted at 8 ml from a Superose 6 column HR 10/30. The end-on views show the donut shape of the cavity formed by CCT subunits; side views show the chaperonin double-ring structure with the characteristic striated barrel shape. The electron density corresponding to the mass of the oligomers bound to CCT is labelled with asterisks. To aid interpretation of electron microscopy images, cartoons (right) show end-on and side views of a model of CCT (pdb 4B2T) bound to a voluminous oligomer, depicted as a cylinder.

The results were analysed using StavroX software^32^, which identified four intermolecular crosslinks involving CCT and α-syn A53T at a false discovery rate (FDR) < 0.05 (Fig. 4b, Table 1). All CCT residues were located inside the CCT cavity. CCTζ-K5, CCTζ-K10 and CCTγ-K380 were situated close to one another (CCTζ and CCTγ are adjacent subunits^33^,^34^), whereas CCTβ-K527 is on the opposite surface of the chaperonin cavity). Ten additional crosslinked intermolecular peptides were identified at a FDR of <0.072 (Supplementary Table S1), five of which originated from CCTζ and CCTγ. Although the score and FDR are insufficient for complete reliability, nine peptides were located within the CCT cavity; this is unlikely to be due to random crosslinking, as similar numbers of Lys and Ser residues are located in- and outside the cavity. These findings suggest a specific interaction between residues within the CCT cavity and the soluble α-syn A53T oligomers, mainly through CCTζ and CCTγ subunits. In parallel, the largest complexes (eluted at 8 ml) were analysed by TEM. Although the oligomer heterogeneity did not allow a more detailed structural study, a direct observation of CCT end-on and side views showed large structures bound inside and/or near the CCT cavity (Fig. 5).

The fact that most of the oligomers could not be completely encapsulated and could therefore impair CCT lid closure led us to question whether they might inhibit CCT ATPase activity. Our results indicated that CCT maintained the same ATPase activity regardless of the absence or presence of oligomers (3.15 ± 1.37 μM ATP/min^−1^μM^−1^ CCT for CCT and 3.08 ± 1.3 μM ATP/min^−1^μM^−1^ CCT, respectively). This could imply that the complex dissociation rate allows ATP hydrolysis before CCT binds again to the α-syn A53T oligomers, but also that CCT could hydrolyse ATP, regardless of the presence of a voluminous substrate within the cavity.

### CCT inhibits α-syn A53T oligomer cytotoxicity in cell cultures

**Figure 6:**
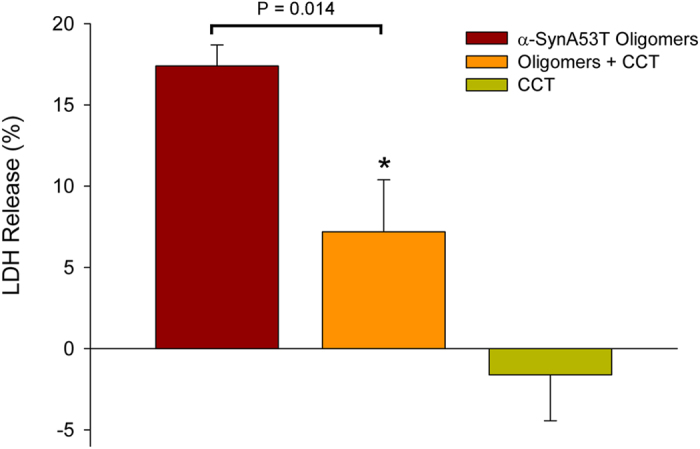
CCT abolishes α-syn A53T oligomer cytotoxicity in SH-SY5Y cells. Mean values ( ± SEM) for LDH release from SH-SY5Y cells treated with CCT (*n* = 5), α-syn A53T oligomers (*n* = 6) and the mixture of both proteins (*n* = 6) compared with solubilized cells (100%). Significant differences within the values of oligomers and oligomers bound to CCT were obtained using Student’s t-test (P = 0.014).

The interaction of molecular chaperones with amyloid proteins is not only able to inhibit fibrillation, but also neutralizes their toxicity^13^,^26^,^35^,^36^. α-syn oligomeric species are currently thought to be the primary toxic species leading to neurodegeneration in a pathological state^37^. Moreover, extracellular α-syn oligomers, which are elevated in cerebrospinal fluid (CSF) of PD patients^38^,^39^, are taken up by neuronal cells and to cause neurotoxicity^40^,^41^. We therefore assayed CCT ability to abolish exogenous α-syn A53T oligomer cytotoxicity in cell cultures. SH-SY5Y neuroblastoma cells were treated with purified α-syn A53T oligomers, CCT, or a mixture of both (15 h). Cytotoxicity was then measured by assaying lactate dehydrogenase (LDH) release from the cells; whereas CCT was not toxic, the oligomers were moderately toxic (Fig. 6). When α-syn A53T oligomers were pre-incubated with CCT, their toxicity decreased significantly (Fig. 6). This finding implies a role for CCT in inhibiting α-syn A53T fibre formation and α-syn A53T oligomer cytotoxicity.

## Discussion

**Figure 7:**
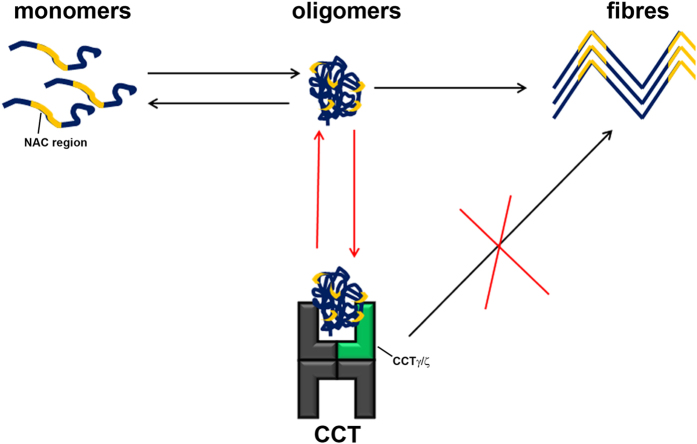
CCT mechanism of α-syn A53T fibre assembly. Through its γ and ζ subunits, CCT interacts with the α-syn NAC region when assembled in soluble oligomers. This interaction allows partial encapsulation of oligomers and inhibits their conversion to amyloid fibres, and cytotoxicity.

Pathological aggregates such as those found in amyloid-related medical disorders appear to form through a common process that involves protein misfolding and formation of transient, soluble oligomers, which in turn give rise to amyloid fibres. These fibres share a core architecture, the “cross-β” structure, in which the β-strands are perpendicular to the fibre axis and generate β-sheet arrays oriented parallel to this axis^42^. α-synuclein, whose aberrant misfolding and aggregation is responsible for Parkinson’s disease, is an intrinsically disordered, 140-residue protein that is divided into three regions; an N-terminal with high-helical propensity (residues 1–61), a central hydrophobic region (NAC; residues 61–95), and a very negatively charged, unstructured C-terminal region (residues 95–140). The amyloid formation process in α-syn begins by protein misfolding and formation of soluble oligomers, constituted by a core of non-amyloid antiparallel β-sheet structure^43^ (nucleation step). These oligomers then convert to fibres^44^, which elongate by monomer addition^45^ (elongation step). Our data show that the eukaryotic chaperonin CCT, a molecular chaperone involved in the folding of many important proteins, interacts specifically with soluble oligomers of α-syn A53T and inhibits their evolution to fibres (Fig. 7) as well as their intrinsic toxicity (Fig. 6). This could be physiopathologically and therapeutically relevant, as extracellular α-syn oligomers, currently believed to be the most neurotoxic species, are internalized by neuronal cells and lead to neurotoxicity^40^,^41^.

CCT interaction with α-syn A53T oligomers is consistent with the low, sub-stoichiometric CCT concentration (1:100) needed to inhibit fibre assembly. As the concentration of soluble oligomers in the course of the fibrillation reaction is very low (3–15% of total protein)^46^,^47^,^48^, and there are between 10 and 100 monomers per oligomer^48^, at a 1:100 molar ratio of CCT:α-syn A53T there is in fact an excess of chaperonin to oligomers, sufficient to bind forming oligomers and halt the amyloid fibre assembly reaction before elongation. Inhibition of α-syn fibre formation by interaction with fibrillation intermediates is also used by other chaperones such as αB-crystallin^49^, Hsc70^35^, Hsp90^26^ and 14-3-3η^36^.

Our results show that CCT interacts with the α-syn NAC region, which would satisfy requirements for CCT binding. This chaperonin interacts with substrates that host a mixture of nonpolar and polar residues in their binding sites^10^, as is the case of the α-syn NAC region. α-syn NAC interaction with molecular chaperones is also described for Hsp70 and Hsp90^25^,^29^,^50^. The NAC region is an important part of the α-syn oligomeric core, and our results and those of others show that it is also a major participant in fibre formation (Fig. 2)^51^. Interaction with CCT would thus shield this region and inhibit fibrillation. The α-syn acidic C terminus does not form part of the oligomeric core and remains unfolded^43^. It is not completely solvent-exposed, however, and is suggested to be partially packed against other protein regions and to participate in overall oligomer structure^52^. This would explain our results, which show less CCT inhibition of fibre assembly of the α-syn A53T mutants that lack the C-terminal part (α-syn A53T ΔC and NAC) than of those with full-length α-syn A53T (Fig. 2). Two possibilities are suggested, that 1) deletion of the C terminus increases the number of exposed NAC domains in the oligomers and therefore, the number of CCT molecules needed to shield them, or 2) the CCT recognition surface in α-syn A53T oligomers is a combination of NAC and C-terminal residues.

Our XS-MS experiments confirmed a specific interaction between CCT and soluble α-syn A53T oligomers in the interior of the chaperonin cavity. The α-syn A53T residues crosslinked with CCT subunits are situated in the N-terminal rather than the NAC or C-terminal regions, probably because the N-terminal region has 10 of the 14 Lys residues in the protein. Only K80 is present in the NAC domain, which might not be exposed in the oligomeric structure. In the chaperonin, three of the four most reliable CCT:α-syn crosslinks (and 5 of the 10 with a less reliable signal) originate from Lys and Ser residues located in the CCTζ and CCTγ subunits, which are adjacent in each of the CCT rings^33^,^34^; they could thus form an interacting surface with the α-syn soluble oligomers in the cavity interior. The identification of α-syn-interacting subunits could be essential for future therapeutic uses. The CCTα subunit interacts with huntingtin, and overexpression of this subunit and exogenous delivery of a truncated CCTα version modulate the Huntington disease cell phenotype^13^,^53^. Exogenous addition of CCT ζ and γ subunits could therefore be of interest as a future therapy that would block the toxicity of extracellular α-syn oligomers, which are high in cerebrospinal fluid (CSF) of Parkinson patients^38^ and can promote prion-like spreading^41^.

Most oligomers that form during the aggregation reaction are too large to be completely encapsulated by CCT, which can accommodate up to ~70 kDa proteins (~5 α-syn A53T monomers) in its ~10 × 10 nm cavity. Our TEM experiments showed a 10 nm width for the oligomeric structures, in accordance with TEM and small angle X-ray scattering (SAXS) studies showing that α-syn oligomers are ellipsoid structures 10 nm wide and 12–18 nm long^37^,^46^,^54^. CCT would thus only partially encapsulate the α-syn A53T oligomers, and complete encapsulation of the substrate by closure of the cavity induced by ATP binding and hydrolysis would not form part of the CCT-mediated inhibition of α-syn fibre formation. TEM imaging analysis of the crosslinked complexes between CCT and the largest oligomers supports this hypothesis (Fig. 5). The figure shows a gallery of end-on and side views; whereas the former show large α-syn A53T oligomers in contact with CCT, in the latter the oligomers appear to protrude from the chaperonin cavity. This incomplete encapsulation would explain the nucleotide-type-independent inhibition of fibre assembly and the absence of oligomer modification by CCT, and suggest a mechanism different other than protein folding, which requires ATP hydrolysis and substrate enclosure. Although this is not the canonical mode of action of CCT for substrate folding, it is a common mechanism for CCT inhibition of fibrillation and toxicity of amyloid proteins. CCT inhibits huntingtin amyloid fibre assembly and toxicity via a nucleotide-independent mechanism^11^,^13^. Indeed, the isolated apical domain of CCT1 (CCTα), which can bind substrates but not encapsulate or fold them, modulates mutant huntingtin cell phenotypes^53^,^55^. We can thus assume that the functional role of the interaction with huntingtin and α-syn is the inhibition of toxicity. CCT interaction with amyloid proteins would sequester the toxic species, hindering their deleterious action. The subsequent fate of these toxic species is still unaddressed. We could speculate that, as chaperones act in a concerted manner, they could later be transferred to other proteins of the proteostasis network for processing. For example, this interaction would promote *in vivo* oligomer clearance by oligomer transfer to Hsp70 and subsequent degradation via CHIP (C terminus of HSC70-interacting protein) and the ubiquitin-proteasome pathway^56^. Future *in vivo* experiments focussed on proteostasis networks will be needed to completely understand this type of cytotoxicity inhibition mechanism.

The ATPase-independent effect of CCT in α-syn and huntingtin fibrillation is the only similarity between the two mechanisms. Our results demonstrate that there is a distinct mechanism for huntingtin and α-syn amyloid assembly inhibition. The CCTα subunit binds to a huntingtin amphipathic helix in monomers^18^ and small fibres tips^27^. The interaction with small oligomers (<70 kDa) is also suggested by cryo-elecron microscopy^27^. Furthermore, the CCT concentration must be almost equimolar (1:1–1:10 molar ratio CCT:huntingtin) to block amyloid formation efficiently^11^,^27^. This suggests that CCT binds preferentially to low molecular weight species and/or there is a low affinity interaction. In contrast, CCT inhibits α-syn A53T even at low substoichiometric concentrations, and the chaperonin uses CCTζ and CCTγ (rather than CCTα) subunits to interact with the NAC region only in oligomers. In the course of the fibrillation reaction, this region evolves from unfolded (monomers) to a non-amyloid antiparallel β sheet (oligomers)^43^ and finally, to an amyloid parallel β sheet^57^. CCT interaction is thus NAC conformation-dependent.

In conclusion, our results here combined with previous studies with huntingtin indicate a general CCT function in the control of amyloidopathies, which should prompt new studies of the role of this eukaryotic chaperonin, alone or in cooperation with other chaperones, in the control of fibrillation and toxicity of other amyloid-related proteins. A detailed study of the mechanism used by the chaperonin would be essential, as we show that CCT uses specific subunits and distinct modes of inhibition for each amyloid-related protein.

## Materials and Methods

### Protein cloning, overexpression and purification

The mutation A53T was incorporated in the human α-syn wt-encoding vector using the QuickChange method. Deletions were produced using the method of Liu *et al*.^58^. The α-syn NAC deletion mutant sequence (α-syn 57–102), containing the NAC region (61–95) plus flanking charged residues to increase its solubility^59^, was cloned into the pET28a expression vector (Novagen) digested with *Nde*I and *Xho*I.

The α-syn wt and the A53T and ΔN variants were expressed in *Escherichia coli* BL21 and purified as described^60^, with some modifications. The cell pellet was resuspended in 10 mM Tris–HCl (pH 8.0), 1 mM EDTA, 1 mM PMSF, and lysed by sonication. This suspension was centrifuged (23,500 ×g, 1 h, 4 °C) in a SS-34 rotor (Thermo Fischer), the supernatant boiled (20 min), and centrifuged again as before. Supernatant was collected, precipitated with 60% ammonium sulphate (3 h, 4 °C) and centrifuged again (22,000 ×g, 30 min, 4 °C; SS-34 rotor). The pellet was resuspended in 25 mM Tris–HCl (pH 8.0), loaded onto a 5 ml HiTrap Q-Sepharose column on an ÄKTAFPLC (GE Healthcare), and α-syn was eluted with a 0 to 600 mM NaCl gradient. Fractions containing the protein were concentrated by ultrafiltration and loaded onto a Superdex-200 16/30 chromatography column. The eluted protein was concentrated and stored at −80 °C. α-syn NAC was expressed and purified as described^59^. α-syn ΔC was expressed and purified as for α-syn wt, although using SP-Sepharose resin. Protein concentrations were estimated from absorbance at 275 nm with an extinction coefficient of 5605 M^−1^cm^−1^ for α-syn wt and A53T, 4200 M^−1^cm^−1^ for ΔN and 1400 M^−1^cm^−1^ for ΔC. The NAC concentration was calculated by amino acid analysis.

Bovine testis CCT was purified as described^8^. GroEL was kindly provided by Dr. Arturo Muga (Universidad del País Vasco/Euskal Herriko Unibertsitatea, Bilbao, Spain).

### *In vitro* fibrillation of α-synuclein

Fibre formation was promoted by mixing 100 μl of 35 μM α-syn A53T, wild type or the deletion mutants, alone or with CCT (molar ratio 1:10, 1:50 or 1:100 CCT:α-syn), in 25 mM Tris pH 7.4, 5 mM MgCl_2_, 150 mM NaCl, 10% glycerol, 50 mM KCl, 0.35 mM SDS, 1 mM dithiothreitol (DTT) and 1 mM ADP (CCT buffer) or 10 mM ATP (10 h, 37 °C, with vigorous shaking). When using an ATP regeneration system, we added 1 mM ATP, 20 mM phosphoenolpyruvate (PEP) and 15 μg/ml pyruvate kinase.

### Thioflavin T (ThT) assays

Aliquots (5 μl) were removed from the fibrillation reaction at various times and diluted in 145 μl of 40 μM ThT in 50 mM glycine buffer pH 8.5. Fluorescence was measured immediately at 37 °C with a Hitachi F700 fluorimeter (440 nm excitation/490 nm emission, slits 4 nm).

### Fibre isolation

To obtain fresh fibres, the fibrillation reaction was initially followed by ThT fluorescence and terminated at the beginning of the elongation step (20–40% of usual maximum fluorescence). Fibres were purified as described^61^, with minor modifications. Triton X-100 (1%) was added to the samples and incubated (15 min) to dissolve soluble oligomers. Fibres were subsequently purified from the monomers by ultracentrifugation (141,000 ×g, 60 min, 15 °C). Fibres in the pellet were washed by resuspension and ultracentrifugation in CCT buffer with 1% Triton X-100, and washed twice in the same buffer without detergent. Finally, fibres were resuspended in CCT buffer and stored at 4 °C for up to 24 h. The presence of fibres was evaluated by the ThT assay and by TEM.

### Preparation of α-syn A53T oligomers

α-syn A53T oligomers were prepared as described^30^. Aliquots (250 μl) of 2 mM α-syn A53T were incubated (overnight, 37 °C, without shaking). The solution was centrifuged (14,000 ×g, 15 min, room temperature) to remove large aggregates, and the supernatant was filtered by centrifugation through 100 kDa filters to remove monomeric and small oligomeric species. The unfiltered material was diluted with PBS and refiltered. The process was repeated several times until no protein was detected in the filtrate. Finally, the oligomers were concentrated to a volume of 80 μl and used immediately.

### Co-sedimentation experiments to measure fibre and chaperonin interactions

Purified fibres (3.5 μM at monomer concentration) were incubated with 0.7 μM CCT, GroEL or closed-lid CCT in 25 mM Tris pH 7.4, 5 mM MgCl_2_, 150 mM NaCl, 10% glycerol (30 min, room temperature). When closed-lid CCT was used, AlF_3_ was included. The mixture was centrifuged (141,000 xg, 60 min, 4 °C) and pellets resuspended in buffer to the same volume as the supernatant. Equal amounts of supernatant and resuspended pellet volumes (usually 10 μl) were analysed by SDS-PAGE. Proteins were visualized by Coomassie blue staining and quantified using ImageJ software (US National Institutes of Health). As controls, chaperonins and fibres alone were centrifuged; as the chaperonins did not precipitate at this speed, the percentage of binding was calculated as the percentage of pellet protein signal relative to that of the total protein (pellet + supernatant).

### CCT-ADP-AlF_3_ complex formation

CCT was incubated with ADP-AlF_3_, which mimics the ATP hydrolysis transition step and thus maintains the lid closed, using the protocol by Reissman *et al*.^62^. Lid closure was tested by a proteinase K protection assay, by adding 20 μg/ml proteinase K to the solution, incubation (10 min, 25 °C), followed by SDS-PAGE.

### SEC experiments

α-syn A53T soluble oligomer (1 mg at monomer concentration) was incubated with 0.3 mg oligomeric CCT in 40 μl PBS (30 min, room temperature) and 30 mM ATP when needed, then loaded onto a Superose 6 HR 10/30 column. Fractions were analysed by SDS-PAGE.

### DTSSP crosslinking assays

α-syn A53T soluble oligomer (1 mg) was incubated with 0.3 mg oligomeric CCT in 40 μl PBS (30 min, room temperature), after which 5 mM DTSSP (Thermo Scientific) was added and the mixture incubated. After 30 min, 50 mM Tris was added to stop the crosslinking reaction. The crosslinked complex was purified by SEC using Superose 6 HR 10/30 resin. Presence of the complex was confirmed by SDS-PAGE in reducing conditions, and crosslinking efficiency was examined by non-reducing SDS-PAGE (Supplementary Fig. S6). Fractions 10 and 11 were pooled for the XL-MS experiments.

### Trypsin digestion, LC-MS/MS analysis and peptide identification

Purified complexes were precipitated using the methanol/chloroform method and the protein pellet was dissolved in 10 μl 8 M urea, 25 mM ammonium bicarbonate. After vigorous vortexing, the urea concentration was reduced to 2 M by addition of 30 μl of 25 mM ammonium bicarbonate, followed by 100 ng trypsin (proteomics grade; Sigma-Aldrich); digestion was allowed to proceed at 37 °C for 5 h. The resulting peptide mixture was speed-vac dried and redissolved in 0.1% formic acid.

For LC-MS/MS analysis, we used a nano-LC Ultra HPLC (Eksigent) coupled online with a 5600 triple TOF mass spectrometer through a nanospray III ion source (both from AB Sciex) equipped with a fused silica PicoTip emitter (10 μm × 12 cm; New Objective). The HPLC setup included an Acclaim PepMap 100 trapping column (100 μm × 2 cm, 5 μm particle size; Thermo Scientific) and an Acquity UPLC BEH C18 column (75 μm × 150 mm, 1.7 μm particle size; Waters). Solvents A and B were 0.1% formic acid in water and 0.1% formic acid in acetonitrile, respectively. Peptides were fractionated at a 250 nl/min flow rate at 40 °C in gradient elution conditions consisting of 2% solvent B for 1 min, a linear increase to 30% B in 109 min, a linear increase to 40% B in 10 min, a linear increase to 90% B in 5 min and 90% B for 5 min. The ion source was operated in positive ionization mode at 150 °C with a potential difference of 2300 V. Each acquisition cycle included a survey scan (350–1250 m/z) of 250 ms and a maximum of 25 MS2 spectra (100–1500 m/z).

For peptide identification, PeakView 1.2 (AB Sciex) was used to convert raw MS/MS data to an mgf file that was searched against a custom-made database containing the sequences of human α-syn and of different bovine CCT subunits. The MS/MS ion search was performed using StavroX 3.5.1^32^ with the following settings: trypsin as enzyme allowing 2 and 3 missed cleavages for Arg and Lys residues, respectively, oxidation of Met as variable modification, DSP/DTSSP as crosslinker, and MS and MS/MS tolerances of 20 ppm. Peptide identifications were filtered at a FDR ≤ 5% corresponding to a score ≥133.

### ATPase activity

CCT (7 μM) was incubated (1 h, 25 °C) alone or with 1.8 mM α-syn A53T oligomers (monomer concentration) to form the complexes. To measure ATPase activity, CCT samples were diluted seven-fold in a reaction mixture containing 50 mM Tris-HCl, 10 mM MgCl_2_, 100 mM KCl, 0.2 mM NADH, 2 mM phosphoenolpyruvate, 15 μg/ml pyruvate kinase, and 30 μg/ml lactate dehydrogenase, pH 7.5^63^. ATP hydrolysis was coupled with NADH reduction, and the resulting decrease in absorbance at 340 nm was measured in a Spectramax spectophotometer. The activity rate was determined from the slope of absorbance *vs* time plot, considering the NADH extinction coefficient (6,220 M^−1^cm^−1^).

### LDH release cytotoxicity assays

SH-SY5Y cells were cultured in DMEM:F12 medium supplemented with 10% iFBS (heat-inactivated foetal bovine serum), glutamine and antibiotics (37 °C, 5% CO_2_). Cells were seeded for 24 h at a density of 2 × 10^4^ cells/well in a 96-well plate (100 μl culture medium), to which 10 μl α-syn A53T oligomers (800 μM at monomer concentration), 7 μM CCT, or a mixture of both (800 μM α-syn A53T oligomers, 7 μM CCT, pre-incubated 1 h to assure complex formation) were added and incubated (15 h). Controls included cells to which detergent was added (100% cell death), untreated cells, or buffer alone. Cytotoxicity was evaluated using the CytoTox 96 kit (Promega) to measure the activity of lactate dehydrogenase (LDH), a stable cytosolic enzyme released following cell lysis. Values are shown as a percentage of total detergent-treated lysed cells after subtraction of values for buffer alone.

### Electron microscopy

Samples were visualized by negative staining in a transmission electron microscope. Aliquots were applied to 400 mesh grids (Maxtaform Cu/Rh HR26) coated with a thin (8 nm) carbon layer and glow-discharged for 20 s. Grids were stained (2 min) with 2% uranyl acetate and air-dried before visualization. Images were acquired using a JEM 1200 (JEOL) electron microscope operated at 100 kV and recorded on Kodak-electron SO-163 film. The diameter of oligomeric structures was measured using ImageJ. Images of CCT-α-syn A53T oligomers were acquired using a JEM 1010 (JEOL) microscope and recorded using a 4K×4K TemCam-F416 (TVIPS) digital camera. Single particles were selected manually and extracted from micrographs using the XMIPP software package^64^.

## Additional Information

**How to cite this article**: Sot, B. *et al*. The chaperonin CCT inhibits assembly of α-synuclein amyloid fibrils by a specific, conformation-dependent interaction. *Sci. Rep.* **7**, 40859; doi: 10.1038/srep40859 (2017).

**Publisher's note:** Springer Nature remains neutral with regard to jurisdictional claims in published maps and institutional affiliations.

## Supplementary Material

Supplementary Information

## Figures and Tables

**Table 1:** Residues involved in intermolecular crosslinks.

CCT subunit	Crosslinked	
	Residue 1 (CCT)	Residue 2 (SynA53T)
CCTβ	K527	K45
CCTγ	K380	N-terminal
CCTζ	K5	K12
CCTζ	K10	K60
