# Supplementary material for: The chaperonin CCT inhibits assembly of α-synuclein amyloid fibrils by a specific, conformation-dependent interaction
Source: Sci Rep. 2017 Jan 19;7:40859. doi: 10.1038/srep40859 (PMC5244355; doi:10.1038/srep40859)
Supplement: Supplementary Information [file srep40859-s1.pdf]

The chaperonin CCT inhibits assembly of α-synuclein amyloid fibrils by a specific, conformation-dependent interaction

Begoña Sot[1,2,*], Alejandra Rubio-Muñoz[1,2], Ahudrey Leal-Quintero[1,†], Javier Martinez-Sabando[1], Miguel Marcilla[3], Cintia Roodveldt[4], José M. Valpuesta[1,*]

Supplementary Figures

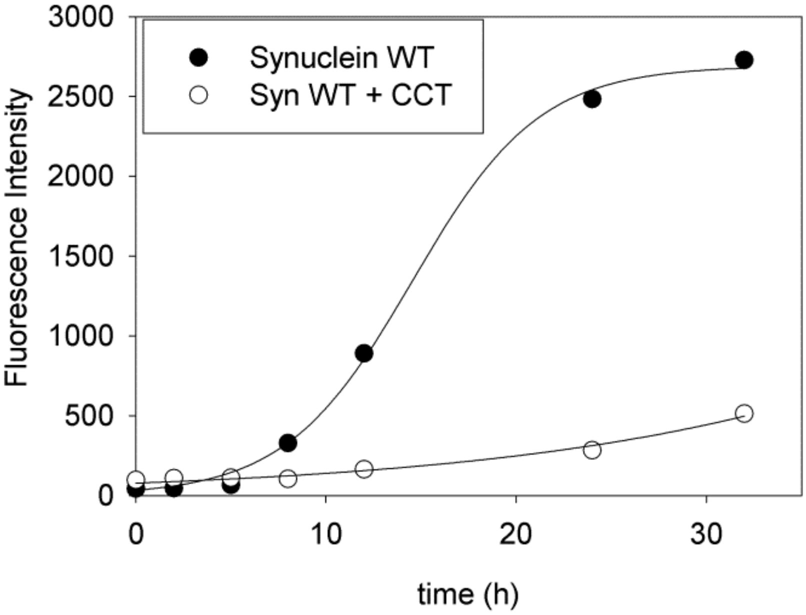

Figure S1. CCT inhibits α-syn wild type fibrillation. (A) Fibrillation kinetics of α-syn WT alone and with CCT (1:10 CCT:α-syn A53T molar ratio), as measured by ThT fluorescence. CCT alone was used a control, and showed no significant fluorescence increase. The fluorescence data is normalized as a percentage of the maximum fluorescence obtained. A representative experiment is shown.

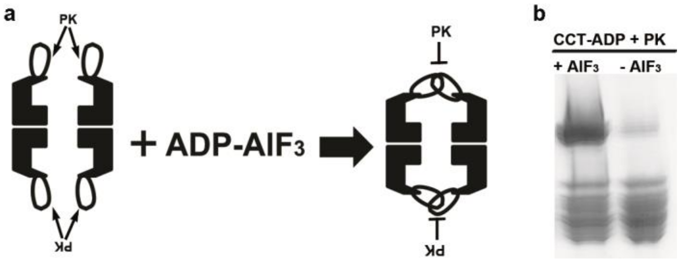

Figure S2. The CCT-ADP-AlF$_3$ complex maintains the CCT lid closed. (**a**) Scheme showing lid movements after binding to ADP-AlF$_3$; proteinase K (PK) cleavage sites in the lid are indicated. (**b**) The closure of the CCT cavity was tested using proteolysis protection. The lid of CCT monomers can be cleaved by proteinase K. Closure of the chaperonin lid is induced and stabilized by CCT incubation with ADP-AlF$_3$**,** and is therefore protected from proteolysis.

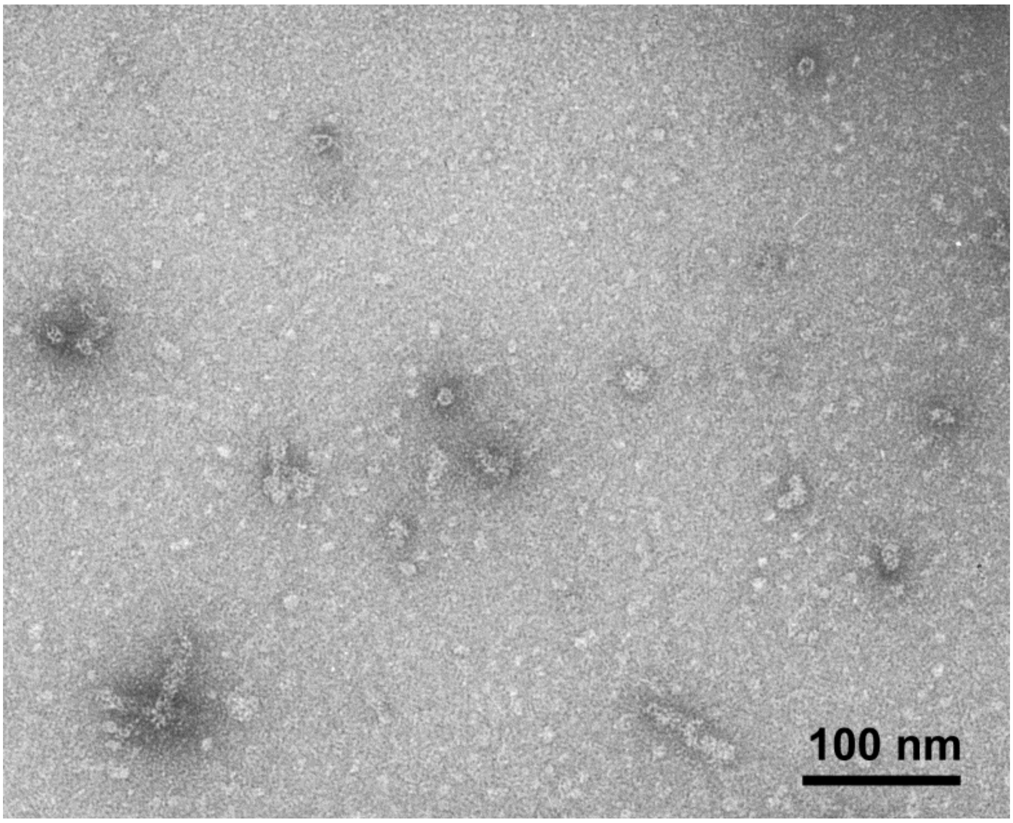

Figure S3. TEM images of α-syn A53T oligomers

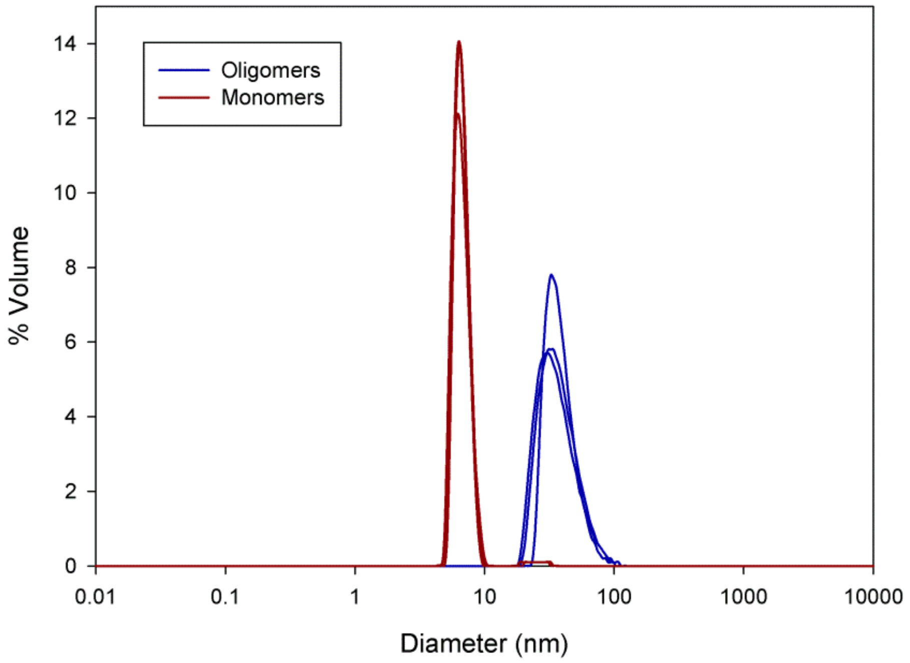

Figure S4. Dynamic light scattering (DLS) experiments of α-syn A53T monomers (red) and oligomers (blue). Size distribution of the molecules present, expressed as a percentage of volume.

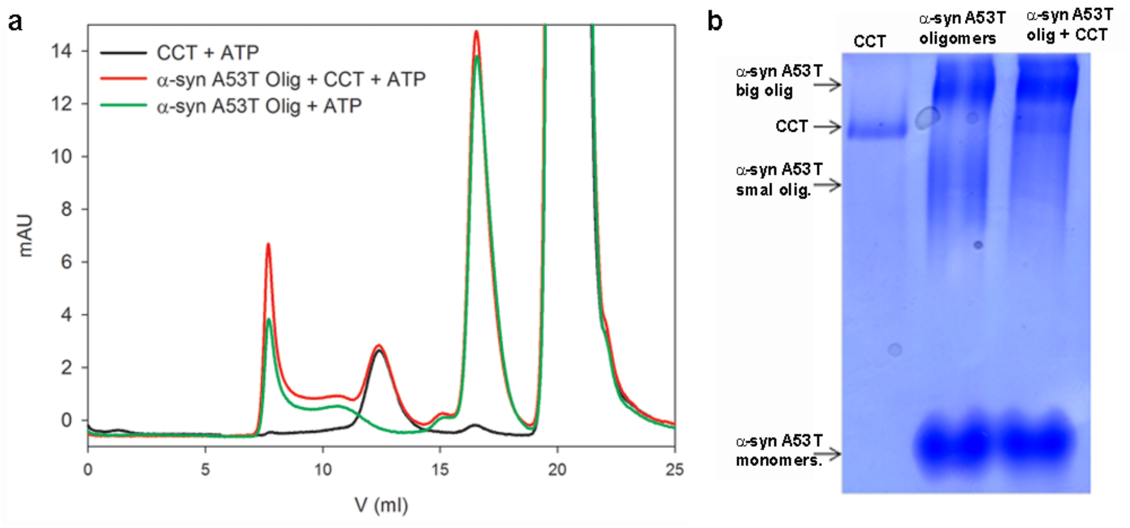

Figure S5. Effect of CCT and ATP in α-syn A53T oligomers. (a) SEC analyses of CCT, α-syn A53T oligomers and the mixture of both, preincubated 1h with 30 mM ATP before loading. Oligomers eluted at 8 and 10 ml, CCT at 12 ml, monomers at 17 ml. The peak at 20 ml corresponds to the nucleotide. **(b)** Native 5.5% acrilamide gels of CCT, α-syn A53T oligomers and the mixture of both, pre-incubated 1h with 30 mM ATP before loading.

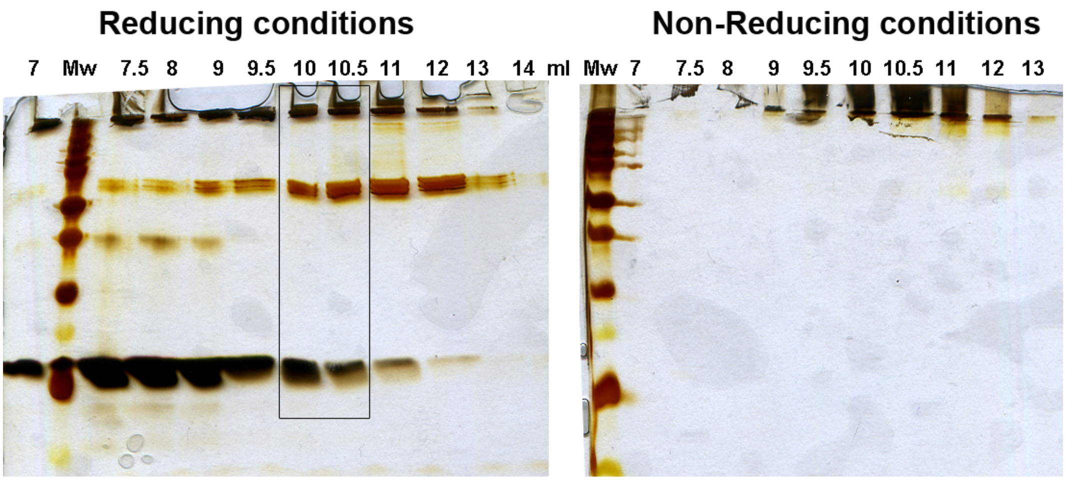

Figure S6. Silver-stained SDS-PAGE showing crosslinked CCT-α-syn A53T oligomer complexes after elution from Superose 6 in reducing and non-reducing conditions. Each lane is labelled with the elution volume (ml). Mw, molecular weight markers. In non-reducing conditions (right), the crosslinked proteins did not enter the gel. In reducing conditions (left), DTSSP was reduced and the individual proteins visualized, showing that the complex is present. Fractions eluted at 10 and 10.5 ml (black rectangle) were analysed by LC-MS/MS.

Table S1. Detailed information of intermolecular crosslinked peptides identified

Score	m/z	z	Dev(ppm)	Peptide(1)	Protein(1)	From(1)	To(1)	Peptide(2)	Protein(2)	From(2)	To(2)	Site(1)	Site(2)	Rank	FDR	Inside Cavity
290	1134.909	3	-19.73	{MAAVKTLNPK]	CCTζ	0	10	[TKEQVTNVGGAVVTGVTAVAQK]	α-Synuclein	59	80	K10	K2	1	0.000	Yes
222	580.310	4	12.24	[AKEGVVAAAEK]	α-Synuclein	11	21	{MAAVKTLNPK]	CCTζ	0	10	K2	K5	1	0.000	Yes
169	754.349	3	0.16	{MDVFMK]	α-Synuclein	0	6	[GASKEILSEVER]	CCTγ	378	389	{0	K4	1	0.000	Yes
161	1036.883	3	-10.39	[VDNIIKAAPRKR]	CCTβ	517	528	[TKEGVVHGVTTVAEK]	α-Synuclein	44	58	K11	K2	1	0.044	Yes
117	708.67	3	10.42	[EGVLYVGSK]	α-Synuclein	35	43	[VQAEHSNSK]	CCTζ	466	474	S8	S8	2	0.054	No
115	589.049	4	5.63	[mVINHLEK]	CCTθ	55	62	[TKEGVLYVGSK]	α-Synuclein	33	43	K8	K11	2	0.063	Yes
98	580.813	4	-5.42	[MIGIKK]	CCTη	194	199	[GLSKAKEGVVAAAEK]	α-Synuclein	7	21	K6	K6	1	0.067	Yes
95	560.903	5	-15.68	[SVTLLIKGPNK]	CCTζ	371	381	[GLSKAKEGVVAAAEK]	α-Synuclein	7	21	K11	K4	1	0.07	Yes
92	682.657	3	-6.96	[EGVVAAAEK]	α-Synuclein	13	21	[mVINHLEK]	CCTθ	55	62	K9	K8	2	0.072	Yes
92	910.208	4	-0.21	[ILIANTGmDTDK]	CCTβ	237	248	[TKEQVTNVGGAVVTGVTAVAQK]	α-Synuclein	59	80	K12	K2	3	0.072	Yes
